# Impact of COVID-19 Lockdown on Non-Alcoholic Fatty Liver Disease and Insulin Resistance in Adults: A before and after Pandemic Lockdown Longitudinal Study

**DOI:** 10.3390/nu14142795

**Published:** 2022-07-07

**Authors:** Ángel Arturo López-González, Bárbara Altisench Jané, Luis Masmiquel Comas, Sebastiana Arroyo Bote, Hilda María González San Miguel, José Ignacio Ramírez Manent

**Affiliations:** 1 Faculty of Dentistry, University School ADEMA, 07009 Palma, Balearic Islands, Spain; angarturo@gmail.com (Á.A.L.-G.); s.arroyo@eua.edu.es (S.A.B.); h.gonzalez@eua.edu.es (H.M.G.S.M.); 2 Investigation Group ADEMA SALUD IUNICS, 07003 Palma, Balearic Islands, Spain; 3 IDISBA, Balearic Islands Health Research Institute Foundation, 07004 Palma, Balearic Islands, Spain; lmasmiquel@gmail.com (L.M.C.); jignacioramirez@telefonica.net (J.I.R.M.); 4 General Practitioner Department, Balearic Islands Health Service, 07003 Palma, Balearic Islands, Spain; 5 Faculty of Medicine, University Balearic Islands, 07009 Palma, Balearic Islands, Spain

**Keywords:** COVID-19, non-alcoholic fatty liver disease, insulin resistance

## Abstract

Background: Non-alcoholic fatty liver disease is a chronic disease caused by the accumulation of fat in the liver related to overweight and obesity, insulin resistance, hyperglycemia, and high levels of triglycerides and leads to an increased cardiovascular risk. It is considered a global pandemic, coinciding with the pandemic in 2020 caused by the “coronavirus disease 2019” (COVID-19). Due to COVID-19, the population was placed under lockdown. The aim of our study was to evaluate how these unhealthy lifestyle modifications influenced the appearance of metabolic alterations and the increase in non-alcoholic fatty liver disease. Methods: A prospective study was carried out on 6236 workers in a Spanish population between March 2019 and March 2021. Results: Differences in the mean values of anthropometric and clinical parameters before and after lockdown were revealed. There was a statistically significant worsening in non-alcoholic fatty liver disease (NAFLD) and in the insulin resistance scales, with increased body weight, BMI, cholesterol levels with higher LDL levels, and glucose and a reduction in HDL levels. Conclusions: Lockdown caused a worsening of cardiovascular risk factors due to an increase in liver fat estimation scales and an increased risk of presenting with NAFLD and changes in insulin resistance.

## 1. Introduction

Non-alcoholic fatty liver disease (NAFLD) is a chronic disease that can be asymptomatic. It is caused by an accumulation of fat in the liver that is not related to alcohol consumption [1] but that is related to overweight and obesity [2,3], insulin resistance [4], states of hyperglycemia [3,5], and high levels of triglycerides in the blood. As such, it is also related to metabolic dysfunction [4,6,7,8] and increased cardiovascular risk factors [9,10].

In its advanced stages, this pathology can cause non-alcoholic steatohepatitis (NASH) [11], which is characterized by necrosis and inflammation with a rapid progression to fibrosis and cirrhosis [3], causing liver failure and the appearance of other liver pathologies^5^ such as hepatocellular carcinoma [4]. These pathologies do not occur in the initial stages of NAFLD [9,12]. However, it can also favor the appearance of other diseases, affecting organs and leading to an increase in morbidity and mortality [3]. There are different scales to detect the prevalence and risk of developing NAFLD by determining different clinical parameters without the need to resort to invasive diagnostic techniques. However, one of the most useful tools to determine the presence of this alteration with a high degree of certainty as well as its complications when it is in an advanced phase is to perform a liver ultrasound as well as to take liver biopsies to obtain a positive result and to determine a pathology showing organ damage [1,11,13].

In the last few years, there has been an increase in the prevalence of NAFLD worldwide [2,9], but especially in Western countries due to changes in lifestyle and eating patterns as well as a tendency towards a sedentary lifestyle. In some countries such as the United States, it is the most common cause of liver disease [11,13].

This increased prevalence has caused NAFLD to be considered a global pandemic [14], coinciding with the pandemic caused by a virus of the *Coronaviridae* family called SARS-CoV-2 in 2020 [15]. The disease caused by this virus became known internationally as “coronavirus disease 2019” (COVID-19) and consists of respiratory and gastrointestinal symptoms [16]. It was found that patients with underlying diseases had a higher risk of COVID-19 infection and a worse prognosis in the case of infection [14,17].

The rapid spread and severity of the COVID-19 pandemic became a threat to public health, and measures such as population lockdowns were put in place [18]. In Spain, this was established by the Royal Decree 463/2020 of 14 March, which declared a state of emergency [19].

This state of lockdown had a negative impact on physical and mental health, with a decrease in physical activity [20,21] and lifestyle modifications with unbalanced nutritional patterns [22,23,24], causing an increase in sedentary lifestyle [22] and greater rates of overweight and obesity [25], favoring the appearance of diseases [26,27,28] and metabolic alterations such as NAFLD and metabolic syndromes, leading to a rise in cardiovascular risk factors [17].

It is important to avoid the development of these pathologies, which are usually asymptomatic in their initial stages and altered in clinical and laboratory tests when they have progressed and therapeutic measures and when lifestyle changes to stop them may no longer be effective [9,13].

Our objective was to evaluate how these unhealthy lifestyles due to lockdown influenced different anthropometric and clinical parameters as well as the increase in the prevalence of presenting NAFLD through risk scales in a sample of Spanish workers, with the aim of taking the appropriate preventive measures to reduce their secondary effects and the development of other diseases.

## 2. Materials and Methods

We carried out a prospective study in workers from the Autonomous Community of the Balearic Islands and the Valencian Community. These workers are people who attended periodic occupational medical check-ups between March 2019 and March 2021. Active workers between 18 and 69 years of age who agreed to participate in the study after signing the corresponding informed consent form we included.

A total of, 6283 workers were selected, 47 of whom were excluded (19 of them for not wanting to participate in the study and the remaining 28 because they did not attend the second examination), so the study was carried out on a final sample of 6236 workers.

Inclusion criteria:

− Aged between 18–69 years and an active worker;
− Healthy without diseases that do not allow the medical check-up to be passed;
− Belong to one of the companies collaborating in the study;
− Agreement to participate.

During the medical examination, the assessment of the anthropometric measurements and extractions for analysis were carried out by the health personnel of the occupational health units. Previously, the uniformity in the collection of said measures was standardized, homogenized, and compared to all of the health personnel.

Weight (kilograms) and height (centimeters) measurements were taken with an SECA 700 model measuring rod with a maximum weight capacity of 200 kg as well as an SECA 220 telescopic measuring rod with millimeter divisions and ranging in size from 60 to 200 cm. To measure height, the person was barefoot and standing with their back resting on the stadiometer with their feet together and with their hands and feet at the sides of their body while looking forward with their eyes and ears in the Frankfort plane.

When measuring the abdominal perimeter of the waist, it was necessary to use an SECA 20 model tape measure with a length between 1 and 200 cm. The measurements were taken by placing the tape parallel to the ground at the level of the last floating rib. The patient had to be placed in an upright position with their feet together and with their abdomen relaxed together with their arms resting at both sides of the body. For the hip circumference, an SECA 200 model tape with a length between 1 and 200 cm was used. The patient should stood in the previous position, but in this case, the tape measure was passed horizontally at hip height.

By dividing the waist circumference by the height and the hip circumference, the waist/height and waist/hip ratios were obtained. The cut-off points were 0.50 for the first index in both men and women and 0.85 for the second index in women and 0.95 in men [29].

To control blood pressure, the patient had to be placed in the supine position, and an OMRON M3-type calibrated automatic sphygmomanometer was used. Three measurements were made at intervals of one minute in order to calculate the mean value.

To carry out the blood tests, a peripheral venous puncture was performed after a 12 h fasting period, and they were subsequently processed at 48–72 h. Automated enzymatic methods were used to measure parameters such as cholesterol, glucose, and triglycerides. The results of the analyzed parameters were expressed in mg/dl. The determination of the HDL was performed by precipitation with dextran sulfate. The values were also expressed in mg/dl. LDL levels, also expressed in mg/dl, were calculated using the Friedewald formula (LDL = total cholesterol–HDL–triglycerides/5).

A patient was considered obese when their BMI was greater than 30 [29], which was calculated by dividing weight by height in meters squared. The determination of the percentage of the body’s insulin resistance was analyzed with the following scales:

- The metabolic score for insulin resistance (METS-IR), which is a mathematical approach to quantify hepatic sensitivity to insulin using fasting parameters [30].
  ⚬ METS-IR = Ln [(2FPG) + TG] × BMI)/(Ln[HDLc]).
- The triglyceride glucose index (TyG), which is used for the presumptive diagnosis of insulin resistance [30].
  ⚬ TyG = Ln [fasting TG (mg/dL) × FPG (mg/dL)/2].
- The triglyceride glucose index–body mass index, which is a useful marker for insulin resistance in non-diabetic individuals (TyG-BMI) [30].
  ⚬ TyG-BMI = TyG × BMI.
- The triglyceride glucose index–waist to height ratio (TyG-WtHR) [30].
  ⚬ TyG-WtHR = TyG × WtHR.
- Triglyceride glucose index–waist circumference (TyC-WC) [30].
  ⚬ TyG-WC = TyG × WC.

Scales to determine non-alcoholic fatty liver disease:

- Lipid accumulation product (LAP) [30].
  ⚬ Men: LAP = (waist circumference (cm) − 65) × (triglyceride concentration (mMol)).
  ⚬ Women: LAP = (waist circumference (cm) − 58) × (triglyceride concentration (mMol)).
- Fatty liver index (FLI) [30].
  ⚬ FLI = (log (triglycerides) × 10^0.953^ + 0.139 × BMI + 0.71 × log (ggt) + 0.053 × waist circumference − 15.745)/(1 + log (triglycerides) × 10^0.953^ + 0.139 × BMI + 0.718 × log (ggt) + 0.053 × waist circumference − 15.745) × 100.
- Hepatic steatosis index (HSI) [30].
  ⚬ HSI = 8 × ALT/AST + BMI (+ 2 if type 2 diabetes yes, + 2 if female).

A smoker was considered to be a person who had regularly consumed at least one cigarette/day in the previous month or who had had stopped smoking less than a year before. 

Physical activity was determined by the International Physical Activity Questionnaire (IPAQ) [31], a seven-question self-administered questionnaire that assesses the type of physical activity performed in the previous seven days.

### 2.1. Statistical Analysis

A descriptive study was carried out using the different categorical variables by calculating both the frequency and distribution. For the analysis of the quantitative variables, the mean and standard deviation were determined, and for the qualitative variables, the percentage was obtained. For the bivariate analysis, the X2 test (with Fisher′s exact correction if necessary) and Student′s t test were used when the samples were independent. In the multivariate analysis, the binary logistic regression test was used with the Wald method, calculating the odds ratio with a 95% confidence interval and applying the Hosmer–Lemeshow goodness-of-fit test. Statistical analysis was performed with SPSS 28.0 (IBM, New York, NY, USA), accepting a statistical significance level of 0.05.

### 2.2. Ethical Considerations and Aspects

The study was approved by the Clinical Research Ethics Committee of Balearic Islands Health (Aproval Code: IB 4383/20). The participants received the information regarding the study and signed the informed consent before being included in the study. All procedures were performed in accordance with the ethical standards of the institutional research committee and with the 2013 Declaration of Helsinki.

## 3. Results

Lockdown began for all participants on March 2020, and post-lockdown anthropometric measurements were carried out by the same health personnel from the different health units. Of the 6283 workers who attended the check-ups, 51.9% were female and 48.1% male, constituting a proportional representation of both sexes. The final number of participants was the same each year, representing a total of 6236 Spanish workers. 

Table 1 shows the statistically significant differences in the mean values of the anthropometric and clinical parameters before and after lockdown due to the COVID-19 pandemic and the percentage of women (51.9%) and men (48.1%) who participated in the study.

An increase in body weight and in BMI values can be seen as well as an increase in the abdominal and hip circumference and percentage of body fat with respect to pre-lockdown and post-lockdown values. 

Regarding clinical parameters, an elevation in the liver profile parameters stands out, with a statistically significant increase in transaminases (AST, ALT) and in GGT being observed.

In the other clinical parameters, an increase in triglyceride and total cholesterol levels can be seen, with a rise in LDL cholesterol and a reduction in HDL cholesterol levels.

Systolic blood pressure was affected, and upon comparing blood pressure levels during lockdown, there was a tendency to higher diastolic blood pressure levels. Glucose levels also increased during lockdown, similar to the previously analyzed parameters. 

In relation to the qualitative variables, there was a statistically significant 11% reduction in physical activity as well as a 2% increase in smoking. This indicates that during the months of confinement, both men and women adopted a more sedentary lifestyle, probably due to the restrictive measures imposed by the authorities.

In order to assess the pre-lockdown and post-lockdown differences according to the characteristics of the population, we have stratified it into four of the pathologies of greatest interest in our study: obesity, type 2 diabetes, dyslipidemia, and metabolic syndrome.

In all of them, we can see that there has been an increase in its prevalence over the years and determined that there is a close relationship between all of them because as the number of individuals in the four pathologies increases, when one of them occurs, it is easily associated with the appearance of another.

Although other factors could obviously exist, we believe that lockdown may have had a great influence on obtaining these results.

Upon analyzing the scales for insulin resistance (METS-IR, TyG index, TyG index-BMI, TyG index–waist circumference, TyG index–waist to height ratio) and NAFLD (lipid accumulation product, fatty liver index, hepatic steatosis index), a statistically significant increase in the mean results of all of them is observed during lockdown. If we focus on the pre-lockdown and post-lockdown differences, the worsening of all of the percentages of the different scales studied stands out, with the lipid accumulation product scale being the worst: going from values of 8.30% pre-lockdown to values of 20.40% post-lockdown; the fatty liver index scale also stood out, with an increase of 12.59% compared to pre-lockdown values, as can be seen in Table 2.

Table 3 assesses the changes in the prevalence of the different values of the insulin resistance and non-alcoholic fatty liver scales analyzed pre-lockdown and post-lockdown due to the COVID-19 pandemic, revealing statistically significant results with a difference of more than 2% between the years before and after pandemic in both groups as well as between patients with type 2 diabetes and non-diabetic patients. 

However, the highest variations in both groups are found in the lipid accumulation product scale and in the fatty liver disease scale, with a greater worsening in the NAFLD prevalence scales compared to the insulin resistance scales. It is noteworthy that all of the formulas experienced greater worsening in the non-diabetic group compared to in the diabetic group. The worst values of the different analyzed scales occurred in the metabolic score for insulin resistance (METS-IR) followed by the lipid accumulation product scale and the fatty liver disease scale. These alterations are affected in the same order in diabetic patients; however, their percentage scores are much lower.

**Figure 1:**
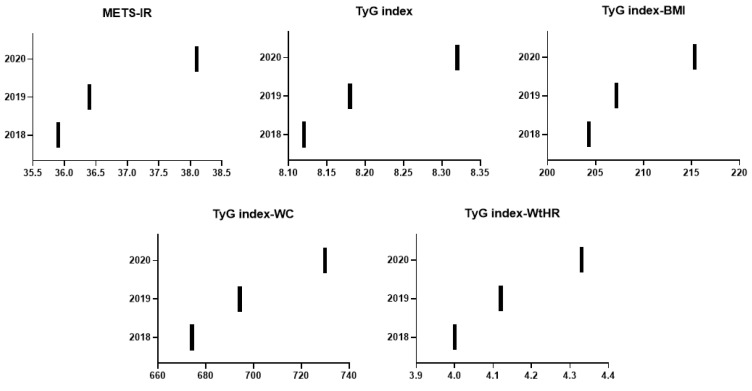
Changes in insulin resistance scales in 2018, 2019, and 2020. METS-IR: metabolic score for insulin resistance; TyG: triglyceride glucose index; BMI: body mass index; WC: waist circumference; WtHR: waist to height ratio.

In Figure 1, we can observe the fluctuation in the different insulin resistance assessment scales during 2018, 2019, and 2020, which would correspond to pre-lockdown and post-lockdown times.

Analyzing the different graphic results, it can be observed that in all of the scales, there was an increase in the values compared to the cut-off points for normality and to the previous years, which would indicate an increased risk of presenting insulin resistance in non-diabetic people as well as a greater risk of developing NAFLD. If we focus on the values for 2020, these present a greater increase compared to the pre-lockdown era due to the changes in lifestyle resulting from it.

**Figure 2:**
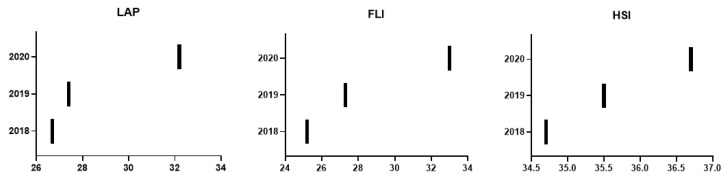
Changes in NAFLD scales in 2018, 2019, and 2020. LAP: Lipid accumulation product; FLI: fatty liver index; HIS: hepatic steatosis index.

By analyzing the NAFLD scales and comparing the values corresponding to pre- and post-lockdown, an increase in the values of the scales analyzed (LAP, FLI, HSI) stands out, as observed in Figure 2, which indicates a greater probability and risk of presenting with NAFLD. This increased risk of NAFLD is caused by the worsening of the parameters that make up the analyzed scales.

Table 4 shows the association and relationship that exists between the different scales analyzed for insulin resistance and non-alcoholic fatty liver disease. We detected a strong correlation, with values close to unity between the METS-IR scale and those for non-alcoholic fatty liver disease, which indicates that the metabolic syndrome is closely and directly related to non-alcoholic fatty liver disease and alterations in its parameters and that it will also cause alterations in the other scales in a statistically significant way. The scales of the TyG index BMI and TyG index waist are also related in a statistically significant and strong way, which shows that when different scales that measure insulin resistance appear with altered parameters, they can also produce an increase in the parameters of the scales for fatty liver.

## 4. Discussion

In recent years, the population has changed its lifestyle, becoming more sedentary, and have changed their eating and sleeping patterns and physical activity, leading to an unhealthy lifestyle. The months of lockdown caused by the COVID-19 pandemic have resulted in several effects and complications on people′s health, and not only those derived from infection with the virus [32]. The state of lockdown had a negative impact on the health and lifestyle of the population [33].

There was a reduction in physical activity, an increase in smoking, and a worsening of dietary habits [33], factors that were aggravated due to lockdown, causing an increase in the rate of obesity and overweight, as seen in Table 1. In the study by Cicero et al. [34] and Khan et al. [35], it can be observed that during lockdown, the population increased its consumption of foods rich in carbohydrates, leading to an increase in obesity due to a worsening in the quality of their diet and a decrease in physical activity [34,35].

These changes affected the health of healthy people and aggravated existing chronic pathologies and also caused the appearance of new diseases, increasing cardiovascular risk factors and metabolic diseases [36,37,38].

Anthropometric, clinical, and laboratory tests were affected as well as all body systems [39,40], as can be seen in our study carried out on a Spanish working population, with a statistically significant increase in all of the parameters: weight, BMI, waist circumference, hip circumference, waist to height ratio, waist to hip ratio, body fat percentage, blood pressure, basal glycemia, total cholesterol, LDL cholesterol, and triglycerides, together with a significant decrease in HDL cholesterol. Liver enzymes also showed a progressive elevation over the years, which, although far from abnormal levels, is statistically significant, as shown in Table 1.

The new lifestyles derived from the lockdown have caused a statistically significant increase in obesity and overweight, which can be quantified with an increase in weight, BMI, percentage of fat mass, and abdominal perimeter as well as alterations in the lipid profile, simultaneously leading to a worsening of the obesity pandemic and an increase in metabolic pathologies [41,42] such as metabolic syndrome, insulin resistance in both the diabetic and non-diabetic individuals, and a greater risk of developing NAFLD [43], as seen in Table 2 and Table 3, all of which affect the adult population worldwide [44,45], with metabolic syndrome representing the most frequent chronic liver disease in the United States [46].

Low HDL cholesterol, a large waist circumference, hypertriglyceridemia, hyperglycemia, and hypertension are components of metabolic syndrome, a global measure of cardiovascular disease [47,48]. All of these parameters were also affected in our study. Lifestyle modifications caused a reduction in HDL cholesterol, an increase in total cholesterol and LDL cholesterol as well as triglyceride levels, and an increase in anthropometric parameters that are related to metabolic syndrome, indicating that this pathology increased its prevalence during lockdown. 

The development of endocrine–metabolic diseases produces increased cardiovascular risk and therefore a higher risk of presenting acute or chronic cardiovascular events as well as other pathologies such as insulin resistance and NAFLD [9,49]. Both entities can be asymptomatic in the initial stages [9], but there are different scales and formulas (METS-IR, TyG, TyG-BMI, TyG-WC, TyG-WtHR, LAP, FLI, HIS) that can help to detect it, with the objective of diagnosing it in its early stages and attempting to stop its progression [30]. 

In clinical practice, insulin resistance refers to a state in which a given insulin concentration is associated with a subnormal glucose response to endogenous and/or exogenous insulin. This occurs more in association with obesity but can be due to many other causes [50,51]. The presence of insulin resistance together with obesity or overweight can cause the appearance of new pathologies, increasing cardiovascular risk [51,52,53]. Some of the diseases derived from this are the appearance of type 2 diabetes mellitus, fasting hyperglycemia, vascular diseases, metabolic syndrome, polycystic ovary syndrome, NAFLD, and neoplasms, among others [54]. A diagnosis of insulin resistance is made through clinical parameters, many of which are related to metabolic syndrome: hyperglycemia, dyslipidemia, abdominal obesity, and hypertension [50].

In our study, we objectified how there was an increase in insulin resistance levels during lockdown, which was determined by the worsening of the analysis scales related to this entity, as detailed in Figure 1, as well as alterations in the different anthropometric and clinical parameters analyzed, in which a statistically significant increase in the variables studied also stands out (Table 1). All of this caused an increase in the appearance of the metabolic syndrome and other endocrine–metabolic diseases, factors observed in other studies, such as the one by Martinez-Ferran M et al. [55]. This worsening in insulin resistance and increase in metabolic syndrome also resulted in a higher risk of complications in the case of COVID-19 infection [56,57] as well as being obese or overweight [50,58].

As can be seen in Figure 1; Figure 2, it can be observed that in 2018–2019, the values of these scales had a tendency to increase their levels and therefore increase the rate of these pathologies, which indicates that previous to the lockdown, the population already had unhealthy lifestyles. Compared to 2020, we can observe an exponential worsening in all of the scales analyzed, both in the risk of presenting NAFLD and for insulin resistance, in a statistically significant way. These changes indicate that the lifestyles of the population during lockdown were unhealthy and that there was an increase in cardiovascular risk due to these endocrine–metabolic alterations observed in our study, with a worsening in the parameters analyzed on the different scales. 

These alterations are affected in the same order in diabetic patients; however, their percentage scores are much lower in the different scales. This may suggest that the non-diabetic population was more affected by lockdown, perhaps because the diabetic patient, due to their diabetic education, tried to maintain a better diet, as seen in Table 3.

A positive correlation was observed between the alterations in the insulin resistance and metabolic syndrome scales and those for non-alcoholic fatty liver disease, as seen in Table 4.

The study by Cinque et al. [59] shows how changes in lifestyle with changes in diet and physical activity are risk factors for the development of cardio-metabolic diseases [59,60], parameters that were also observed in our study. 

The study by Ghoneim et al. [61] shows how patients with underlying metabolic pathologies were more susceptible to being infected by COVID-19 as well as to presenting complications in the case of infection and the appearance of other diseases due to having a higher cardiovascular risk [61,62].

These cardiovascular and metabolic complications have caused an increase in morbidity and mortality due to hepatic and extrahepatic causes [45] as well as a greater risk of infection by COVID-19 and a worse prognosis in case of infection and requiring hospitalization [47,63,64]. The direct impact of the virus on the liver is unknown but has been seen in several studies, such as the one from Yoo H et al. [65], and the study by Vranic L et al. [66] showed how patients with liver pathologies had a greater risk and a worse prognosis in the case of infection by COVID-19 as well as a higher risk of the decompensation of other pathologies [65,66]. These hepatic alterations were also detected in our study, as shown in Table 1, which shows alterations in the biochemical parameters in relation to the liver profile, where a statistically significant increase in transaminase levels stands out, indicating that the changes in lifestyle also affected the liver and increased the risk of infection by COVID-19 and its worse prognosis [67].

The greater tendency to overweight and obesity and the appearance or aggravation of metabolic diseases and therefore increased cardiovascular risk [68] was also affected by the proinflammatory state caused by endocrine–metabolic pathologies, which, together with the systemic inflammatory state caused by COVID-19, could be the cause of the increased morbidity and mortality [69,70,71].

With the parameters and results described above, we can affirm that lockdown caused a statistically significant deterioration in different health parameters, causing a worsening of cardiovascular and metabolic risk factors [72] and the appearance of new chronic pathologies that have resulted in increased morbidity and mortality. This is in agreement with other studies [73]. 

It is important to highlight the effects of lockdown on the health of the population, because due to globalization, we must be on alert in case another global pandemic that requires lockdown occurs. At present, while the COVID-19 pandemic has yet to be resolved, outbreaks of monkeypox have been reported in Spain, North America, Australia, the United Kingdom, and several countries of the European Union, which has put the health authorities on alert to the possibility of a new health crisis caused by a new infection that could cause a new state of lockdown depending on its evolution [74].

Trying to reduce the appearance of these endocrine–metabolic diseases is important, not only because of the risk of aggravating cardiovascular risk factors and diseases, but also because of the increased risk of infection by COVID-19.

## 5. Conclusions

The lockdown by COVID-19 has caused a worsening in different health parameters, with a negative influence on cardiovascular and metabolic risk factors and the appearance of new chronic pathologies.

There has been an increase in the parameters of the scales that estimate the risk of presenting NAFLD as well as of metabolic syndrome and insulin resistance compared to the months before lockdown. 

According to the NAFLD detection scales that estimate the presence of liver fat based on different clinical and anthropometric parameters, as biochemical and anthropometric cardiovascular risk parameters worsen, the risk of presenting NAFLD increases.

The lockdown caused by COVID-19 has caused a change in healthy lifestyle habits and an increase in harmful behaviors (less physical activity, more tobacco) and therefore a worsening in the health of the population. This should put us on alert, as due to the growth of globalization, a new state of lockdown could be necessary if new pandemic diseases appear. It is important to warn the population about the possible deterioration of their health if an unhealthy sedentary lifestyle is followed. 

## Figures and Tables

**Table 1:** Characteristics of the population per year.

N = 6236	2018	2019	2020	
	Mean ± SD	Mean ± SD	Mean ± SD	*p*-Value
Age (years)	41.1 ± 9.9	42.1 ± 9.9	43.1 ± 9.9	<0.001
Weight (kg)	71.7 ± 16.3	72.2 ± 16.4	73.8 ± 16.5	<0.001
BMI (kg/m^2^)	25.1 ± 4.7	25.3 ± 4.7	25.9 ± 4.7	<0.001
Waist circumference (cm)	82.8 ± 14.0	84.6 ± 14.1	87.6 ± 14.1	<0.001
Hip circumference (cm)	98.7 ± 9.4	99.8 ± 9.4	101.5 ± 9.5	<0.001
Waist to Height ratio	0.49 ± 0.08	0.50 ± 0.08	0.52 ± 0.08	<0.001
Waist to Hip ratio	0.84 ± 0.10	0.85 ± 0.09	0.86 ± 0.09	<0.001
Body fat (%)	24.5 ± 9.1	25.3 ± 8.7	26.9 ± 8.8	<0.001
SBP (mmHg)	120.0 ± 16.8	121.3 ± 16.3	124.6 ± 16.3	<0.001
DBP (mmHg)	76.9 ± 10.7	78.2 ± 10.5	82.8 ± 10.6	<0.001
Glycemia (mg/dL)	90.5 ± 16.4	91.9 ± 15.7	95.4 ± 15.8	<0.001
Total cholesterol (mg/dL)	190.7 ± 37.3	194.3 ± 35.3	202.8 ± 35.7	<0.001
HDL-c (mg/dL)	53.9 ± 13.7	53.1 ± 13.4	50.7 ± 13.7	<0.001
LDL-c (mg/dL)	117.4 ± 40.3	121.4 ± 38.5	131.0 ± 39.0	<0.001
Triglycerides (mg/dL)	96.8 ± 79.2	98.7 ± 78.5	105.8 ± 78.9	<0.001
ALT (U/L)	24.1 ± 28.5	25.7 ± 28.7	28.4 ± 28.7	<0.001
AST (U/L)	21.7 ± 15.5	22.7 ± 15.6	24.0 ± 15.7	<0.001
GGT (U/L)	25.8 ± 27.4	26.8 ± 27.4	28.9 ± 27.4	<0.001
	N (%)	N (%)	N (%)	*p*-value
Women	3236 (51.9)	3236 (51.9)	3236 (51.9)	
Men	3000 (48.1)	3000 (48.1)	3000 (48.1)	
Smokers	1176 (18.9)	1202 (19.3)	1302 (20.9)	<0.001
Physical exercise	2732 (43.8)	2600 (41.7)	2044 (32.8)	<0.001
Social class I	3664 (58.8)	3664 (58.8)	3664 (58.8)	
Social class II	812 (13.0)	812 (13.0)	812 (13.0)	
Social class III	1760 (28.2)	1760 (28.2)	1760 (28.2)	
Obesity	846 (13.6)	860 (13.8)	1007 (16.1)	<0.001
Diabetes type 2	86 (1.4)	100 (1.6)	140 (2.2)	<0.001
Dyslipidemia	2361 (37.9)	2470 (39.6)	3234 (51.9)	<0.001
Metabolic Syndrome	463 (7.4)	865 (13.9)	1304 (20.9)	<0.001

BMI: body mass index; SBP: systolic blood pressure; DBP: diastolic blood pressure; HDL: high-density lipoproteins; LDL: low-density lipoproteins; ALT: alanine aminotransferase; AST: aspartate aminotransferase; GGT: gamma-glutamyl transpeptidase.

**Table 2:** Changes in insulin resistance scales and NAFLD scales in 2018, 2019, and 2020.

	2018	2019	2020		Difference 2018–2019	Difference 2019–2020	
	Mean ± SD	Mean ± SD	Mean ± SD	*p*-Value	Value (%)	Value (%)	*p*-Value
METS-IR	35.9 ± (8.6)	36.4 ± (8.7)	38.1 ± 9.1	<0.0001	0.49 (1.36)	1.71 (4.71)	<0.0001
TyG index	8.1 ± 0.7	8.2 ± 0.7	8.3 ± 0.6	<0.0001	0.06 (0.77)	0.13 (1.63)	<0.0001
TyG index-BMI	204.3 ± 44.0	207.2 ± 43.7	215.3 ± 44.2	<0.0001	2.88 (1.41)	8.09 (3.9)	<0.0001
TyG index-Waist circumference	674.2 ± 137.1	694.3 ± 137.6	729.9 ± 137.6	<0.0001	20.12 (2.98)	35.57 (5.12)	<0.0001
TyG index-Waist to height ratio	4.0 ± 0.8	4.1 ± 0.8	4.3 ± 0.8	<0.0001	0.12 (2.97)	0.21 (5.4)	<0.0001
Lipid accumulation product	26.7 ± 28.5	27.4 ± 30.0	32.2 ± 32.8	<0.0001	2.05 (8,30)	5.45 (20.40)	<0.0001
Fatty liver index	25.2 ± 25.9	27.3 ± 26.8	33.0 ± 27.9	<0.0001	2.09 (8.30)	5.70 (20.89)	<0.0001
Hepatic steatosis index	34.7 ± 6.3	35.5 ± 6.2	36.7 ± 6.4	<0.0001	0.84 (2.43)	1.16 (3.27)	<0.0001

METS-IR: metabolic score for insulin resistance; TyG: triglyceride glucose index; BMI: body mass index.

**Table 3:** Changes in prevalence of high values of insulin resistance scales and non-alcoholic fatty liver disease scales in non-diabetic and diabetic people in 2018, 2019, and 2020.

	Year 2018	Year 2019	Year 2020		Difference 2018–2019	Difference 2019–2020	
Non diabetic	n (%)	n (%)	n (%)	*p*-value	value (%)	value (%)	*p*-value
METS-IR high	418 (6.8)	440 (7.2)	627 (10.3)	<0.0001	0.4 (5.9)	3.1 (43.5)	<0.0001
TyG index high	1240 (20.2)	1292 (21.1)	1512 (24.8)	<0.0001	0.9 (4.5)	3.7 (17.5)	<0.0001
Lipid accumulation product high	1376 (22.4)	1499 (24.6)	1974 (32.4)	<0.0001	2.2 (9.8)	7.8 (31.7)	<0.0001
Fatty liver index high	796 (12.9)	900 (14.7)	1169 (19.2)	<0.0001	1.8 (14.0)	4.5 (30.6)	<0.0001
Hepatic steatosis index high	1933 (31.4)	1998 (32.6)	2198 (36.1)	<0.0001	1.2 (3.8)	3.5 (10.7)	<0.0001
Diabetic							
METS-IR high	39 (45.3)	46 (46.0)	70 (50.0)	<0.0001	0.7 (1.5)	4.0 (8.7)	<0.0001
TyG index high	41 (47.7)	48 (48.0)	70 (50.0)	<0.0001	0.3 (0.6)	2.0 (1.2)	<0.0001
Lipid accumulation product high	36 (47.9)	49 (49.0)	74 (52.9)	<0.0001	1.1 (2.3)	3.9 (8.0)	<0.0001
Fatty liver index high	49 (57.0)	57 (57.0)	82 (58.6)	<0.0001	0.0 (0.0)	1.6 (2.8)	<0.0001
Hepatic steatosis index high	75 (87.2)	90 (90.0)	132 (94.3)	<0.0001	2.8 (3.2)	4.3 (4.8)	<0.0001

METS-IR: metabolic score for insulin resistance; TyG: triglyceride glucose index.

**Table 4:** Pearson correlation coefficients between insulin resistance scales and NAFLD scales.

	LAP	FLI	HSI
METS-IR	0.588	0.861	0.879
TyG INDEX	0.653	0.439	0.092
TyG index BMI	0.685	0.892	0.837
TyG index waist	0.772	0.910	0.676
TyG index waist/high	0.761	0.888	0.701

METS-IR: metabolic score for insulin resistance; TyG: triglyceride glucose index; BMI: body mass index; LAP: lipid accumulation product; FLI: fatty liver index; HIS: hepatic steatosis index.

## Data Availability

Data available on request due to restrictions (privacy or ethical).
